# Tinel’s Sign in Peripheral Nerve Lesions

**Published:** 1918-09

**Authors:** 


***Tinel's Sign in Peripheral Nerve Lesions.*** By W. W. Mc Donald. *British Medical Journal*, July, 1918.

Mc Donald found cases where a well marked faradic muscular response was present when the nerve had been completely divided; he also found that the reaction of degeneration does not always follow the section of a nerve. He believes that electrical tests are of great assistance in arriving at a complete conclusion when taken in conjunction with other methods of examination, but they should not be regarded as final, as it is important that other methods of diagnosing the interruption of a nerve should have their true value recognized. He considers that, of these, Tinel's "le signe du fourmillement", or the distant tingling on percussion (D. T. P.), is the most important. This sign depends on the fact that when young axis-cylinders are percussed, there is a sensation of tingling in the cutaneous area corresponding to the ultimate distribution of the axis-cylinders, e. g., if the musculo-spiral nerve is divided in the arm and the proximal end is percussed, no tingling will be felt by the patient. The whole area supplied by the degenerating part will be insensitive. When, later, regeneration occurs as early as 4-6 weeks, tapping over the site of the wound causes the patient to experience a tingling sensation in the skin over the dorsum of the 1st and 2nd metacarpals. When tingling in the hand follows tapping on the arm, this indicates continuity of the neurone between the arm and the brain.

If the new axis-cylinders are arrested or involved in a neurone the portion of the nerve from which D. T. P. can be elicited is never longer than 2-3 cms. and is situated at the site of the lesion. This is what is commonly found in interruption by neuroma or section.

As the axis-cylinders regenerate there is a *pari passu* downward extension of the D. T. P. and in healthy subjects growth takes place at the rate of 1-2 mm. a day.

When new axis-cylinders have completely developed their function, the D. T. P. disappears; this usually takes about 100 days; i. e., every 100 days ten cm. of regenerated nerve will lose its D. T. P. and the next ten cm. will show it. It is thus possible to study the progression of regeneration by this simple test.

This sign always precedes the return of muscular tonus, voluntary movement and normal electrical reactions by a considerable interval; therefore it is of great importance and value after operation for nerve graft or suture.

In cases where there is a nerve lesion in a limb with multiple wounds, the existence of the D. T. P. indicates which wound is responsible for the nerve lesion, and in lengthy wounds it indicates the exact level of the nerve injury.

When D. T. P. can be obtained early in the second month for more than ten cms. below the lesion, one can affirm that the complete recovery will take place in a few months.

In testing the D. T. P., always percuss from the distal end and proceed slowly, otherwise there may be obtained a persistent sensation of tingling. Also, the limb must not be shaken too much for the same reason.

In the elderly, alcoholic, or debilitated subjects, where nerve regeneration is imperfect or altogether fails, D. T. P. does not occur and its complete absence gives a grave prognosis, The writer shares the opinion of others of wide experience, of not operating on nerve lesions till between the 4th and 6th month, except in causalgia where early operation is advisable.

